# Fluctuations of Serum Neuron Specific Enolase and Protein S-100B Concentrations in Relation to the Use of Shunt during Carotid Endarterectomy

**DOI:** 10.1371/journal.pone.0124067

**Published:** 2015-04-10

**Authors:** Marko Dragas, Igor Koncar, Dragan Opacic, Nikola Ilic, Zivan Maksimovic, Miroslav Markovic, Marko Ercegovac, Tatjana Simic, Marija Pljesa-Ercegovac, Lazar Davidovic

**Affiliations:** 1 Clinic for Vascular and Endovascular Surgery, Clinical Centre of Serbia, Belgrade, Serbia; 2 Faculty of Medicine, University of Belgrade, Belgrade, Serbia; 3 Faculty of Health, Medicine and Life Sciences, Department of Physiology, Maastricht University, Maastricht, The Netherlands; 4 Clinic of Neurology, Clinical Centre of Serbia, Belgrade, Serbia; 5 Institute of Medical and Clinical Biochemistry, Faculty of Medicine, University of Belgrade, Belgrade, Serbia; Scientific Inst. S. Raffaele Hosp., ITALY

## Abstract

**Objective:**

To evaluate the changes in serum neuron specific enolase and protein S-100B, after carotid endarterectomy performed using the conventional technique with routine shunting and patch closure, or eversion technique without the use of shunt.

**Materials and Methods:**

Prospective non-randomized study included 43 patients with severe (>80%) carotid stenosis undergoing carotid endarterectomy in regional anesthesia. Patients were divided into two groups: conventional endarterectomy with routine use of shunt and Dacron patch (csCEA group) and eversion endarterectomy without the use of shunt (eCEA group). Protein S-100B and NSE concentrations were measured from peripheral blood before carotid clamping, after declamping and 24 hours after surgery.

**Results:**

Neurologic examination and brain CT findings on the first postoperative day did not differ from preoperative controls in any patients. In csCEA group, NSE concentrations decreased after declamping (P<0.01), and 24 hours after surgery (P<0.01), while in the eCEA group NSE values slightly increased (P=ns), accounting for a significant difference between groups on the first postoperative day (P=0.006). In both groups S-100B concentrations significantly increased after declamping (P<0.05), returning to near pre-clamp values 24 hours after surgery (P=ns). Sub-group analysis revealed significant decline of serum NSE concentrations in asymptomatic patients shunted during surgery after declamping (P<0.05) and 24 hours after surgery (P<0.01), while no significant changes were noted in non-shunted patients (P=ns). Decrease of NSE serum levels was also found in symptomatic patients operated with the use of shunt on the first postoperative day (P<0.05). Significant increase in NSE serum levels was recorded in non-shunted symptomatic patients 24 hours after surgery (P<0.05).

**Conclusion:**

Variations of NSE concentrations seemed to be influenced by cerebral perfusion alterations, while protein S-100B values were unaffected by shunting strategy. Routine shunting during surgery for symptomatic carotid stenosis may have the potential to prevent postoperative increase of serum NSE levels, a potential marker of brain injury.

## Introduction

Carotid endarterectomy (CEA) is well recognized as the best method for stroke prevention in patients with severe symptomatic and asymptomatic carotid artery stenosis [1]. Despite becoming the most common procedure performed in vascular surgery, several aspects of CEA are still under debate, especially those considering the optimal surgical technique (conventional or eversion) and the use of carotid shunting [2,3]. Many surgeons prefer routine shunting [4,5], others use the shunt selectively based on various neuromonitoring techniques [6], while some surgeons perform CEA without shunting [7,8]. Similarly, in the current practice carotid endarterectomy is most frequently performed by eversion or conventional technique, based on the preference, training and experience of the respective surgeon [2]. Regardless of the shunting strategy and technique employed for the procedure, many authors report excellent results of CEA, with very low stroke and mortality rates [2–8].

However, in the past decade a concern has been raised in regard to the subtle, subclinical brain damage during carotid revascularization [9]. In the search for specific biochemical markers of brain injury, analogue to troponin for myocardial ischemia, several compounds have been investigated, with special attention on neuron specific enolase and protein S-100B. Neuron specific enolase (NSE; γγ-enolase) is a glycolytic pathway isoenzyme predominantly present in neurons, which can be promptly released into blood after brain injury [10]. It has a biologic half-life of 20 hours, and is routinely used in clinical practice for prognostication after cardiac arrest [10,11]. Calcium-binding S-100B protein is predominantly expressed and secreted by astroglial cells in the central nervous system, with a short half-life of 2 hours [12]. Albeit the predictive value of these biomarkers was demonstrated in a variety of pathologic insults to the brain such as cardiac arrest [13], subarachnoid haemorrhage [14], and ischemic stroke [15–17], their significance in detecting subtle cerebral damage in patients undergoing surgery has not yet been elucidated. Elevated plasma concentrations of protein S-100B and NSE have been found to correlate with the occurrence of postoperative cerebral dysfunction in patients undergoing coronary and carotid revascularization by several authors [18–20]. In contrast, others found no association between the levels of serum biomarkers and development of subtle or clinically apparent neurologic deficit after carotid endarterectomy [21–24].

The aim of this study was to evaluate the changes in serum NSE and protein S-100B concentrations, after carotid endarterectomy performed with either of the two most commonly used approaches: conventional CEA with routine shunting and patch closure, and eversion endarterectomy without the use of shunt.

## Materials and Methods

Ethical board of the Clinical Centre of Serbia, as well as the Ethics Committee of Belgrade Medical Faculty (decision 440/X-17) approved the study, and all patients gave informed written consent for participation in the trial.

This prospective, non-randomized study was conducted on patients operated for carotid stenosis during a four-month period (September 2012—January 2013) by two consultant vascular surgeons (MD and IK) at a referral, high-volume vascular center, in which CEA is performed either by eversion technique without shunting, or conventional technique with routine shunting and patch closure. Both surgeons underwent similar vascular training (provided by the senior author), and had comparable carotid endarterectomy volumes (>60 procedures per year) and neurologic complication rates (<1.2%) in the past five years. All consecutive patients with significant (>80%) carotid stenosis admitted for surgery by the two participating surgeons were included in the study. Although fully proficient in both surgical techniques commonly employed at our institution, each surgeon had more experience with one of the strategies, and for the purposes of the study was dedicated to it throughout the trial period. Exclusion criteria defined for this study were the following: urgent carotid endarterectomy, carotid restenosis, major surgery (including contralateral carotid endarterectomy) in the past six months, history of cancer, history of brain trauma or surgery, and patient incapacity to undergo surgery under regional anesthesia.

Preoperative evaluation included detailed history, laboratory analysis, carotid Duplex ultrasonography, brain multidetector computerized tomography (MDCT) and examination by an independent neurologist. All operations were performed under regional anesthesia achieved by the combination of superficial and deep cervical plexus block (20ml Marcaine + 14ml Lidocaine), without the use of volatile or intravenous general anesthetics. Neurologic status was assessed by the anesthesiologist during the entire procedure. Before and during carotid clamping blood pressure was medically maintained (phenylephrine, nitroglycerin) at about 20% above baseline levels in all cases. Physiologic monitoring included pulse oximetry, continuous standard ECG and invasive arterial pressure monitoring through the radial artery. Carotid bifurcation was routinely exposed through a longitudinal incision. Upon systemic heparinization (Heparin 100 IU/kg), carotid arteries were clamped for 2 minutes to test the adequacy of cerebral perfusion, and carotid stump pressure was measured. In patients without neurologic alterations during test clamping we proceeded with carotid endarterectomy without declamping. The patients were divided into two groups based on the technical preference of the operating surgeon. One of the surgeons (MD) performed conventional carotid endaretrectomy with the routine use of shunt (Polyshunt, Perouse Medical, Ivry le Temple, France) and Dacron patch (csCEA), while the other (IK) performed eversion carotid endarterectomy without the use of shunt (eCEA). Blood samples were drawn from the peripheral vein to determine the levels of neuron specific enolase and protein S-100B immediately before carotid clamping, after declamping (10 minutes after clamp release, allowing the equilibrium of observed biomarkers in peripheral blood), and 24 hours after surgery. Less than 30 minutes from collection, samples were centrifuged (3000 rpm for 10 minutes) and the serum was stored at -70°C for later analysis. Concetrations of NSE and S-100B were measured with the use of commercially available automated electrochemiluminescence immunoassays (Elecsys 2010, Roche Diagnostics GmbH, Mannheim, Germany). Detection limits for NSE and S-100B were 0.25 μg/L and 0.02 μg/L, respectively.

After surgery, all patients were monitored for 24 hours in the intensive care unit, and underwent follow up neurological examination, as well as brain MDCT on the first postoperative day. Patients with uneventful postoperative course were discharged from the clinic 48–72 hours after surgery and underwent further clinical and ultrasonography follow up through the outpatient clinic.

Statistical analysis was performed using SPSS 17.0 for Windows (SPSS, Chicago, Ill). Based on the published data on specific markers during carotid revascularization. power analysis was performed for changes in serum levels of NSE and S-100B between different time points (before surgery and 24 hours after surgery for NSE, and before surgery and after declamping for S-100B) [20,22,26]. The number of more than 38 participants (19 per group) was found to be sufficient for detection of significant difference of observed parameters within the groups, with the power of at least 0.80 and a significance level of 0.05. Continuous values of observed characteristics were presented by mean with standard deviation, while categorical variables were presented in percentages. Continuous variables between groups were assessed with Student’s t-tests and Mann-Whitney U tests, while the significance of categorical variables between groups was evaluated with chi-square tests and Fisher exact tests. Statistical difference for repeated measurements was assessed with Wilcoxon signed rank test, Friedman test and Kruskal-Wallis test. Post hoc analysis was performed with Dunn's Multiple Comparison Test. All statistical tests were two-sided with the threshold of significance set at P less than 0.05.

## Results

During the trial period the two participating surgeons performed 61 carotid endarterectomies. Eighteen patients were excluded from the study: due to the urgent nature of carotid endarterectomy (3 patients), recent major surgery (7 patients), history of cancer (4 patients), incapacity to undergo surgery under regional anesthesia (2 patients), and refusal to participate in the study (2 patients). Finally, 43 patients met the inclusion criteria and were enrolled in the study. Twenty-one patients were operated with conventional carotid endarterectomy technique with the routine use of shunt and Dacron patch (csCEA group), while 22 patients underwent eversion carotid endarterectomy, without the use of shunt (eCEA group). Majority of patients were male (63%), with an average age of 66.3±7.5 years, and similar distribution of risk factors and comorbid conditions between the study groups (Table 1).

**Table 1:** Demographics, risk factors and comorbid conditions of CEA patients.

Variable	csCEA (n = 21)	eCEA (n = 22)	P
Age (years)	65.29±7.54	67.18±7.60	0.416
Body mass index (kg/m^2^)	24.77±2.51	25.68±3.25	0.322
Male gender	15 (71.4)	12 (54.5)	0.252
Symptomatic carotid disease	9 (42.9)	12 (54.5)	0.547
Arterial hypertension	20 (95.2)	19 (86.4)	0.607
Diabetes mellitus	4 (19.1)	3 (13.6)	0.698
Hyperlipoproteinemia	14 (66.7)	15 (68.2)	1.000
Smoking	16 (76.2)	17 (77.3)	1.000
Coronary artery disease	10 (47.6)	8 (36.4)	0.543
Statin therapy	13 (61.9)	16 (72.7)	0.526
Contralateral carotid occlusion	3 (14.3)	2 (9.1)	0.664
Contralateral stenosis 80–99%	1 (4.8)	2 (9.1)	1.000
Peripheral arterial disease	6 (28.6)	3 (13.6)	0.281

Nine (42.9%) patients in csCEA group and 12 (54.5%) patients in eCEA group sustained symptoms of cerebral ischemia (transitory ischemic attack or minor stroke) in the distribution of the diseased carotid artery within the 30 days preceding surgery, while the remaining patients were asymptomatic (P = ns). The two groups were also comparable in regard to baseline values of biochemical markers for atherosclerosis and inflammation (Table 2).

**Table 2:** Baseline biochemical characteristics of patients undergoing CEA.

Variable	csCEA (n = 21)	eCEA (n = 22)	P
Cholesterol (mmol/L)	5.10±1.08	5.81±1.23	0.052
HDL (mmol/L)	1.04±0.17	1.15±0.23	0.076
LDL (mmol/L)	3.24±0.94	3.73±1.05	0.122
Triglycerides (mmol/L)	1.80±0.78	2.03±1.42	0.840
Apo A1 (mmol/L)	1.23±0.35	1.34±0.19	0.135
Apo B (mmol/L)	0.96±0.28	1.08±0.21	0.097
Apo E (mmol/L)	0.04±0.01	0.04±0.01	0.884
Lp a (mmol/L)	0.36±0.39	0.21±0.29	0.170
hsCRP (mg/L)	4.60±5.82	4.60±4.58	0.927
IL-6 (pg/mL)	19.17±18.31	13.28±18.74	0.375
Homocysteine (mmol/L)	14.26±2.73	15.08±6.05	0.855

Average clamping time in the eCEA group was 28±5 minutes (range 18–38 minutes), while in csCEA group the carotid arteries were clamped in average 4.2±1 minutes (range 3.5–5 minutes), including test clamping, shunt placement and time after shunt removal to the completion of patch anastomosis (P = 0.001). The duration of surgery was significantly longer in csCEA compared to eCEA group (103.7±10.4 minutes vs. 89.2±16.3 minutes; P = 0.001), while the other intraoperative variables were similar between the two groups (Table 3). No patients developed neurologic alterations during carotid clamping, and carotid stump pressure exceeded 45mmHg in all cases, indicating adequate cerebral collateral circulation. No deaths, neurologic, or other complications were recorded in the postoperative period. In all patients neurologic examination findings and brain MDCT scans 24 hours after surgery did not differ from preoperative controls.

**Table 3:** Intraoperative variables of patients undergoing CEA.

Variable	csCEA (n = 21)	eCEA (n = 22)	P
Mean TA at test clamping (mmHg)	109.33±10.86	109.98±11.55	0.851
Mean stump pressure (mmHg)	64.61±19.57	54.61±16.34	0.058
Mean TA during clamping/shunt (mmHg)	110.79±7.48	107.02±8.32	0.126
Mean TA after declamping (mmHg)	107.32±8.70	102.77±8.13	0.084
Clamping time (min)	4.23±0.97	27.91±4.96	**0.001**
Duration of surgery (min)	103.67±10.44	89.23±16.30	**0.001**
Complicated plaque	10 (47.6)	6 (27.3)	0.191

The fluctuations of NSE and protein S-100B were analyzed at different time points within groups, as well as between the two study groups. There were no differences in baseline pre-clamp concentrations of observed parameters between the study groups. Mean baseline levels of NSE and protein S-100B were 9.51±4.23 μg/L and 0.065±0.031 μg/L, respectively.

**Fig 1:**
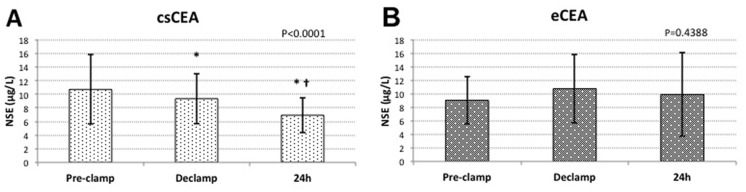
Variations of NSE serum concentrations within groups. **(A)** csCEA group; **(B)** eCEA group; *—significant compared to pre-clamp (P<0.01); † - significant compared to declamp (P<0.05)

In the csCEA group the concentrations of NSE significantly decreased from baseline values after carotid declamping (10.70±5.06 μg/L vs. 9.34±3.66 μg/L; P<0.01), and continued to decline 24 hours after surgery (6.92±2.61 μg/L; P<0.01). However, in the eCEA group NSE concentrations after declamping and 24 hours after surgery were higher than pre-clamp values, without statistically significant difference between time points (9.04±3.50 μg/L vs. 10.76±5.07 μg/L vs. 9.88±6.15 μg/L; P = ns). (Fig 1)

**Fig 2:**
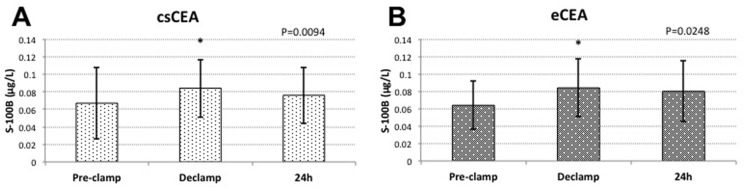
Variations of protein S-100B serum concentrations within groups. **(A)** csCEA group; **(B)** eCEA group; *—significant compared to pre-clamp (P<0.05).

Protein S-100B concentrations significantly increased in both groups upon carotid declamping, decreasing 24 hours after surgery. In the csCEA group measured S-100B concentrations before carotid clamping, after declamping and 24 hours after surgery were 0.067±0.041 μg/L; 0.084±0.033 μg/L*; and 0.076±0.032 μg/L, respectively (*P<0.05). Similarly, in the eCEA group protein S-100B levels were 0.064±0.028 μg/L before clamping, 0.084±0.033 μg/L* after declamping, and 0.080±0.035 μg/L 24 hours after surgery (*P<0.05). (Fig 2)

**Fig 3:**
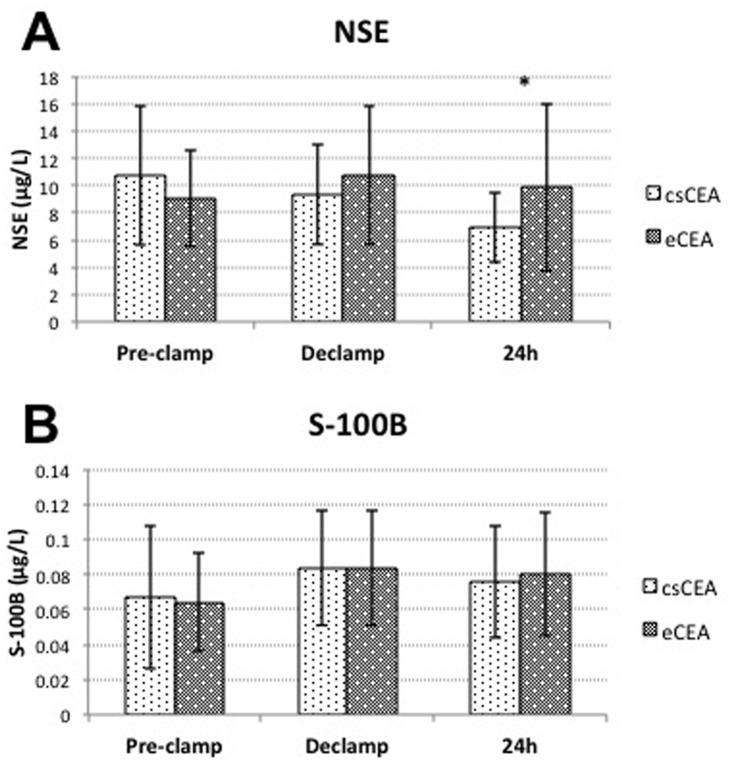
Variations of NSE and protein S-100B serum concentrations between groups. **(A)** variations of NSE concentrations; **(B)** variations of protein S-100B concentrations; *—P<0.01

In the analysis between groups, serum NSE concentrations were found to be significantly lower in the csCEA group compared to eCEA group 24 hours after surgery (6.92±2.61 μg/L vs. 9.88±6.15 μg/L; P = 0.006). The values of observed parameters did not significantly differ between the two groups at other time points. (Fig 3)

**Fig 4:**
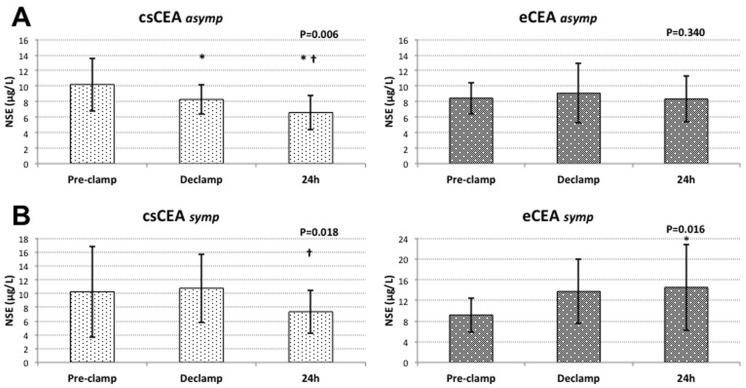
Variations of NSE serum concentrations in relation to the symptomatology of carotid disease. **(A)** asymptomatic patients; **(B)** symptomatic patients; *—significant compared to pre-clamp (P<0.05); † - significant compared to declamp (P<0.05)

Further sub-group analysis was performed according to the symptomatology of carotid disease. There was no difference in pre-clamp NSE concentrations between asymptomatic and symptomatic patients (9.40±2.93 μg/L vs. 9.63±4.88 μg/L; P = ns). In asymptomatic patients operated with the use of shunt, pre-clamp NSE levels (10.19±3.40 μg/L) significantly declined after declamping (8.28±1.91 μg/L; P<0.05) and 24h after surgery (6.59±2.23 μg/L; P<0.01), while in asymptomatic patients undergoing eversion endarterectomy without shunting, there were no significant changes in NSE concentrations at different time points (8.45±2.02 μg/L vs. 9.07±3.83 μg/L vs. 8.33±2.96 μg/L; P = ns). In symptomatic patients shunted during surgery NSE levels also demonstrated a tendency to decline on the first postoperative day (10.25±6.61 μg/L vs. 10.77±4.95 μg/L vs. 7.35±3.13 μg/L*; *P<0.05). However, in symptomatic patients operated without the use of shunt, serum NSE concentrations significantly increased 24 hours after surgery (9.17±3.31 μg/L vs. 13.72±6.16 μg/L vs. 14.52±8.32 μg/L*; *P<0.05). (Fig 4)

**Fig 5:**
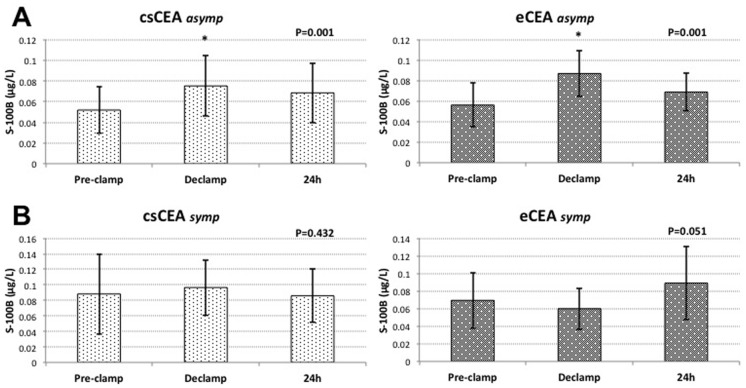
Variations of protein S-100B serum concentrations in relation to the symptomatology of carotid disease. **(A)** asymptomatic patients; **(B)** symptomatic patients; *—significant compared to pre-clamp (P<0.05)

Baseline protein S-100B values were found to be significantly higher in symptomatic patients (0.054±0.021 μg/L vs. 0.078±0.041 μg/L; P = 0.026). In asymptomatic patients S-100B levels significantly increased after declamping, declining on the first postoperative day, in both csCEA (0.052±0.022 μg/L vs. 0.075±0.029 μg/L* vs. 0.068±0.029 μg/L; *P<0.05) and eCEA (0.056±0.021 μg/L vs. 0.087±0.022 μg/L* vs. 0.069±0.018 μg/L; *P<0.05) groups. In symptomatic patients there were no significant differences in protein S-100B concentrations between time points regardless of the shunting strategy (csCEA group: 0.088±0.052 μg/L vs. 0.097±0.036 μg/L vs. 0.086±0.034 μg/L, and eCEA group: 0.069±0.032 μg/L vs. 0.060±0.023 μg/L vs. 0.089±0.041 μg/L; P = ns). (Fig 5)

## Discussion

The results of our study suggest different variations of serum NSE concentrations after carotid endarterectomy in relation to the shunting strategy and endarterectomy technique. Several investigators demonstrated significantly higher baseline NSE values in patients with carotid artery stenosis compared with general population [22,25], similar to our findings. In patients operated with the use of shunt (csCEA group) we noticed a significant decline of NSE levels after declamping and 24 hours after surgery. However, in the non-shunted (eCEA) group NSE concentrations slightly increased, accounting for the significant difference in NSE levels between groups on the first postoperative day. Rasmussen et al. [22] found a significant decrease of initially high NSE values 48 hours after surgery in 20 symptomatic CEA patients, speculating that elevated baseline NSE levels in patients with carotid stenosis (compared with patients operated for abdominal aortic aneurysms) could be the consequence of chronic neuron damage by microembolization, while the subsequent decrease could occur through the elimination of the embolic source. Brightwell et al. [25] recorded a significant postoperative decline of NSE values in patients subjected to carotid artery stenting compared to those undergoing CEA in regional anesthesia with a low shunting rate (10.7%). In this study carotid artery stenting was associated with significantly higher number of embolic signals, but substantially less hemodynamic disturbances, as recorded with trans-cranial Doppler monitoring [25]. Our results also suggest that the fluctuations in NSE values appear to be more sensitive to hemodynamic changes, even in the presence of apparently adequate collateral circulation determined by intact neurologic status of awake patients during cross-clamping, and values of carotid stump pressure. Postoperative decline of high baseline NSE levels in shunted patients could occur due to the early establishment of adequate, unobstructed antegrade blood flow through the treated carotid artery. We can hypothesize that the absence (or delay beyond 24 hours) of this effect in the non-shunted group could be the consequence of subtle perfusion and metabolic disturbances during carotid clamping and reperfusion. Furthermore, our finding of a significant increase in serum NSE concentrations on the first postoperative day only in symptomatic non-shunted patients, could indicate that patients with preexisting brain injury are particularly susceptible to metabolic and oxidative changes caused by relative brain ischemia and subsequent reperfusion.

Changes of protein S-100B concentrations in our study seemed unaffected with the use of shunt or endarterectomy technique, suggesting different release mechanisms for the two potential markers of subtle brain injury. In both groups we noticed a significant increase in S-100B levels after declamping, with a tendency to decline 24 hours after surgery. Similar patterns were also recorded by others [24,26], offering several possible explanations. Intraoperative microembolization during manipulation and dissection of the diseased carotid artery could cause ischemic injury to neural and glial cells accounting for the increase in markers of brain injury. In a recent report, Yoshida et al. [27] found a high incidence (21%) of asymptomatic defects on diffusion-weighted images (DWI) obtained after conventional dissection of the carotid artery during CEA, emphasizing the importance of embolization during carotid surgery. Additionally, correlation between periprocedural embolization during carotid stenting and increase in S-100B levels was demonstrated by several authors [20,25]. In favor of this argument is also our finding of significantly higher pre-clamp S-100B concentrations in symptomatic patients, which could be explained with greater embolic potential of symptomatic carotid plaques. Conversely, Godet et al. [24] found no difference in S-100B values between patients with uneventful postendarterectomy course and overt stroke, concluding that this protein could not serve as a marker of cerebral infarct after CEA in their study. In light of our findings and the absence of postoperative neurological impairment or new lesions on control brain scans in any of our patients, a more likely explanation would be that increased periprocedural embolization can lead to reversible changes in the permeability of blood-brain barrier, allowing for increased temporary leakage of S-100 proteins from cerebrospinal fluid to plasma, as suggested by others [25].

In contrast to our results, Palombo et al. [21] found no differences between NSE and S-100B protein concentrations before and after carotid clamping in patients undergoing CEA with and without the use of shunt. However, blood sampling early after carotid declamping in this study might have easily missed and underestimated the changes in serum concentrations of observed parameters [21].

Although most researchers hypothesize that discrete brain injury during carotid revascularization can occur either due to periprocedural microembolization or hypoperfusion, the role and significance of NSE and protein S-100B in detection and quantification of this injury are still unknown [20,25–29]. Whether the variations in peripheral concentrations of these biomarkers represent irreversible neuronal and glial cell damage, reversible changes in the permeability of blood-brain barrier, or perhaps are a part of protective (“fever-like”) response caused by metabolic flow-related changes, remains unclear [12,25,26].

Limitations of this pilot study prevent us from drawing conclusions in regard to the pathophysiologic mechanisms and clinical relevance of our findings for several reasons. Firstly, having in mind controversial evidence published on the role of specific markers of cerebral injury during CEA, although deemed sufficient for this pilot study, the number of participants would have to be substantially higher in order to evaluate the clinical significance of these findings, preferably with provided direct insight in regional hemodynamic and embolic events by transcranial doppler and diffusion-weighted imaging. Secondly, numerous variations in surgical and anesthetic techniques between centers and surgeons performing CAE, probably decrease external validity of our findings [27–30]. Finally, additional difficulties in comparison of various studies dealing with biomarkers of subtle brain injury during carotid revascularization, arise from diversity of potentially confounding methodological variables, such as patient selection, choice of anesthesia, clamp tolerance, surgical technique, shunting strategy, and methodology of blood sampling [19–27,29–31].

In conclusion, our results suggest different release patterns of NSE and protein S-100B during CEA in regional anesthesia. The variations of NSE concentrations seemed to be influenced by cerebral perfusion alterations during carotid clamping and the use of shunt, even in the presence of adequate collateral brain circulation. Routine use of shunting in symptomatic patients appears to have the potential to prevent the increase of serum NSE concentrations after carotid endarterectomy in regional anesthesia. Further investigations of the cause and significance of these fluctuations, as well as potential metabolic and oxidative cerebral changes during CEA, could have important implications on future clinical practice and choice of the preferential method for carotid revascularization.

## References

[1] Liapis CD, Bell PR, Mikhailidis D, Sivenius J, Nicolaides A, Fernandes J, et al ESVS guidelines. Invasive treatment for carotid stenosis: indications, techniques. Eur J Vasc Endovasc Surg 2009;37: 1–19. 10.1016/j.ejvs.2008.11.006 19286127

[2] AbuRahma AF. Processes of care for carotid endarterectomy: Surgical and anesthesia considerations. J Vasc Surg 2009;50: 921–933. 10.1016/j.jvs.2009.04.071 19660899

[3] AbuRahma AF, Mousa AY, Stone PA. Shunting during carotid endarterectomy. J Vasc Surg 2011;54: 1502–1510. 10.1016/j.jvs.2011.06.020 21906905

[4] Hertzer NR, O’Hara PJ, Mascha EJ, Krajewski LP, Sullivan TM, Beven EG. Early outcome assessment for 2228 consecutive carotid endarterectomy procedures: the Cleveland Clinic experience from 1989 to 1995. J Vasc Surg 1997;26: 1–10. 9240314 10.1016/s0741-5214(97)70139-3

[5] Bellosta R, Luzzani L, Carugati C, Talarico M, Sarcina A. Routine shunting is a safe and reliable method of cerebral protection during carotid endarterectomy. Ann Vasc Surg 2006;20: 482–487. 16639651 10.1007/s10016-006-9037-8

[6] Rerkasem K, Rothwell PM. Routine or selective carotid artery shunting for carotid endarterectomy and different methods of monitoring in selective shunting. Cochrane Database Syst Rev. 2009 10 7;(4):CD000190 10.1002/14651858.CD000190.pub2. 19821268

[7] Radak D, Tanasković S, Matić P, Babić S, Aleksić N, Ilijevski N. Eversion carotid endarterectomy-our experience after 20 years of carotid surgery and 9897 carotid endarterectomy procedures. Ann Vasc Surg 2012;26: 924–928. 10.1016/j.avsg.2011.09.011 22494931

[8] Samson RH, Showalter DP, Yunis JP. Routine carotid endarterectomy without a shunt, even in the presence of a contralateral occlusion. Cardiovasc Surg 1998;6: 475–484. 9794267 10.1177/096721099800600509

[9] Yadav JS. Protecting the brain: How do we measure success? J Am Coll Cardiol 2003;42: 1014–1016. 13678922 10.1016/s0735-1097(03)00900-8

[10] Cooper EH. Neuron-specific enolase. Int J Biol Markers 1994;9: 205–210. 7836797 10.1177/172460089400900401

[11] Sandroni C, Cariou A, Cavallaro F, Cronberg T, Friberg H, Hoedemaekers C, et al Prognostication in comatose survivors of cardiac arrest: An advisory statement from the European Resuscitation Council and the European Society of Intensive Care Medicine. Resuscitation 2014;85: 1779–1789. 25438253 10.1016/j.resuscitation.2014.08.011

[12] Goncalves CA, Leite MC, Nardin P. Biological and methodological features of the measurement of S100B, a putative marker of brain injury. Clin Biochem 2008;41: 755–763. 10.1016/j.clinbiochem.2008.04.003 18454941

[13] Rundgren M, Karlsson T, Nielsen N, Cronberg T, Johnsson P, Friberg H. Neuron specific enolase and S-100B as predictors of outcome after cardiac arrest and induced hypothermia. Resuscitation 2009;80: 784–789. 10.1016/j.resuscitation.2009.03.025 19467754

[14] Oertel M, Schumacher U, McArthur DL, Kastner S, Boker DK. S-100B and NSE: markers of initial impact of subarachnoid hemorrhage and their relation to vasospasm and outcome. J Clin Neurosci 2006;13: 834–840. 16931022 10.1016/j.jocn.2005.11.030

[15] Missler U, Wiesmann M, Friedrich C, Kaps M. S-100 protein and neuron-specific enolase concentrations in blood as indicators of infarction volume and prognosis in acute ischemic stroke. Stroke 1997;28: 1956–1960. 9341703 10.1161/01.str.28.10.1956

[16] Selakovic V, Raicevic R, Radenovic L. The increase of neuron-specific enolase in cerebrospinal fluid and plasma as a marker of neuronal damage in patients with acute brain infarction. J Clin Neurosci 2005;12: 542–547. 15921910 10.1016/j.jocn.2004.07.019

[17] Singh HV, Pandey A, Shrivastava AK, Raizada A, Singh SK, Singh N. Prognostic value of neuron specific enolase and IL-10 in ischemic stroke and its correlation with degree of neurological deficit. Clin Chim Acta 2013;419: 136–138. 10.1016/j.cca.2013.02.014 23438682

[18] Kilminster S, Treasure T, McMillan T, Holt DW. Neuropsychological change and S-100 protein release in 130 unselected patients undergoing cardiac surgery. Stroke 1999;30: 1869–1874. 10471438 10.1161/01.str.30.9.1869

[19] Connolly ES, Winfree CJ, Rampersad A, Sharma R, Mack WJ, Mocco J, et al Serum S100B protein levels are correlated with subclinical neurocognitive declines after carotid endarterectomy. Neurosurgery 2001;49: 1076–1083. 11846900 10.1097/00006123-200111000-00010 PMC3035925

[20] Capoccia L, Speziale F, Gazzetti M, Mariani P, Rizzo A, Mansour W, et al Comparative study on carotid revascularization (endarterectomy vs stenting) using markers of cellular brain injury, neuropsychometric tests, and diffusion-weighted magnetic resonance imaging. J Vasc Surg 2010;51: 584–592. 10.1016/j.jvs.2009.10.079 20045614

[21] Palombo D, Lucertini G, Mambrini S, Zettin M. Subtle cerebral damage after shunting vs non shunting during carotid endarterectomy. Eur J Vasc Endovasc Surg 2007;34: 546–551. 17681825 10.1016/j.ejvs.2007.05.028

[22] Rasmussen LS, Christiansen M, Johnsen J, Gronholdt ML, Moller JT. Subtle brain damage cannot be detected by measuring neuron-specific enolase and S-100B protein after carotid endarterectomy. J Cardiothorac Vasc Anesth 2000;14: 166–170. 10794336 10.1016/s1053-0770(00)90012-0

[23] Sahlein DH, Heyer EJ, Rampersad A, Winfree CJ, Solomon RA, Benvenisty AI, et al Failure of intraoperative jugular bulb S-100B and neuron-specific enolase sampling to predict cognitive injury after carotid endarterectomy. Neurosurgery 2003;53: 1243–1249. 14633290 10.1227/01.neu.0000093493.16850.11 PMC2663381

[24] Godet G, Watremez C, Beaudeux JL, Meersschaert K, Koskas F, Coriat P. S-100 beta protein levels do not correlate with stroke in patients undergoing carotid endarterectomy under general anaesthesia. J Cardiothorac Vasc Anesth 2001;15: 25–28. 11254835 10.1053/jcan.2001.20213

[25] Brightwell RE, Sherwood RA, Athanasiou T, Hamady M, Cheshire NJ. The neurological morbidity of carotid revascularization: using markers of cellular brain injury to compare CEA and CAS. Eur J Vasc Endovasc Surg 2007;34: 552–560. 17719806 10.1016/j.ejvs.2007.06.016

[26] Falkensammer J, Oldenburg AW, Hendrzak AJ, Neuhauser B, Pedraza O, Ferman T, et al Evaluation of subclinical cerebral injury and neuropsychologic function in patients undergoing carotid endarterectomy. Ann Vasc Surg 2008;22: 497–504. 10.1016/j.avsg.2008.01.013 18504102

[27] Yoshida K, Kurosaki Y, Funaki T, Kikuchi T, Ishii A, Takahashi JC, et al Surgical dissection of the internal carotid artery under flow control by proximal vessel clamping reduces embolic infarcts during carotid endarterectomy. World Neurosurg 2014;82: e229–34. 10.1016/j.wneu.2013.06.018 23851209

[28] AbuRahma AF, Stone PA, Srivastava M, Hass SM, Mousa AY, Dean LS, et al The effect of surgeon’s speciality and volume on the perioperative outcome of carotid endarterectomy. J Vasc Surg 2013;58: 666–672. 10.1016/j.jvs.2013.02.016 23601827

[29] Kalimeris K, Kouni S, Kostopanagiotou G, Nomikos T, Fragopoulou E, Kakisis J, et al Cognitive function and oxidative stress after carotid endarterectomy: comparison of propofol to sevoflurane anesthesia. J Cardiothorac Vasc Anesth 2013;27: 1246–1252. 10.1053/j.jvca.2012.12.009 23725684

[30] Wijeyaratne SM, Collins MA, Barth JH, Gough MJ. Jugular venous neurone specific enolase (NSE) increases following carotid endarterectomy under general, but not local anesthesia. Eur J Vasc Endovasc Surg 2009;38: 262–266. 10.1016/j.ejvs.2009.05.011 19540138

[31] Aleksic M, Heckenkamp J, Reichert V, Gawenda M, Brunkwall J. S-100B release during carotid endarterectomy under local anesthesia. Ann Vasc Surg 2007;21: 571–575. 17521874 10.1016/j.avsg.2007.04.002

